# Effect of print layer height on the accuracy and fit of the rapid-prototyped retainers

**DOI:** 10.1590/2177-6709.30.4.e2524250.oar

**Published:** 2025-11-07

**Authors:** Pranita GUPTA, Prabhat Kumar CHAUDHARI, Ritu DUGGAL, Vilas D SAMRIT

**Affiliations:** 1 All India Institute of Medical Sciences, Centre for Dental Education and Research, Division of Orthodontics and Dentofacial Deformities (New Delhi, India).

**Keywords:** Three-dimensional (3D) printing, Clear retainers, Computer-aided design, Accuracy, Orthodontics, SEM (scanning electron microscopy), Impressão tridimensional (3D), Contenções transparentes, Design assistido por computador, Precisão, Ortodontia, MEV (microscopia eletrônica de varredura)

## Abstract

**Introduction:**

The purpose of this study was to comparatively evaluate the accuracy and fit of rapid-prototyped clear retainers printed at two different layer heights (100 µm and 50 µm) using a low-force stereolithography (LFS) 3D printer.

**Methods:**

Forty-two retainers were digitally designed using Maestro 3D Dental computer aided designing (CAD) software (AGE Solutions, Pisa, Italy) and rapid prototyped using an LFS printer (Formlabs Inc, Somerville, MA, USA). Half of the retainers (21) were printed at 50 µm layer height and other half at 100 µm. To assess the dimensional accuracy, linear measurements were taken from the 3D printed retainers using digital caliper (accurate to 0.01 mm) and from the digitally designed retainers using Maestro 3D Dental Studio software. The fit of the retainers was further evaluated with a scanning electron microscope. Accuracy was compared across three groups-digitally designed retainers, and those printed at 50 µm and 100 µm layer height using ANOVA/Kruskal‒Wallis tests. The reliability and internal consistency of the linear measurements were evaluated by the intraclass correlation coefficient (ICC with 95% CI) and Cronbach’s alpha, respectively. Paired t-tests were used to compare the fit of rapid prototyped retainers printed at 100 µm and 50 µm layer heights. Additionally, the printing time for each retainer was recorded.

**Results:**

There was no statistically significant difference (p value > 0.05) between the accuracy of 3D-printed retainers printed at 100 µm and 50 µm layer heights with a high reliability (ICC>0.9) and high internal consistency (Cronbach’s alpha>0.9). For comparative evaluation of the fit, no statistically significant difference (p>0.05) was observed among the retainers printed at 50 µm and 100 µm layer heights, except at the labio-gingival border and at the middle of the incisal edge of the central incisor. Time required for printing retainers at 50 µm is more than that of 100 µm layer height.

**Conclusion:**

Both rapid-prototyped retainers printed at 100 µm and 50 µm layer heights were found to be accurate for clinical use when compared to reference digital retainers with acceptable fit. As the time required for printing retainers at 100 µm is less than that of 50 µm layer height, it is better to use 100 µm layer height for rapid prototyping of the retainers.

## RESUMO

**Introdução:**

O objetivo deste estudo foi avaliar comparativamente a precisão e o ajuste de contenções transparentes de prototipagem rápida impressos em duas alturas de camada diferentes (100 µm e 50 µm) usando uma impressora 3D de estereolitografia de baixa força (LFS).

**Métodos:**

Quarenta e duas contenções foram projetadas digitalmente usando o software de design auxiliado por computador (CAD) Maestro 3D Dental (AGE Solutions, Pisa, Itália) e impressas por prototipagem rápida usando uma impressora LFS (Formlabs Inc, Somerville, MA, EUA). Metade das contenções (21) foram impressas em uma altura de camada de 50 µm e a outra metade, em 100 µm. Para avaliar a precisão dimensional, medições lineares foram feitas nas contenções impressas em 3D usando um paquímetro digital (com precisão de 0,01 mm) e nas contenções projetadas digitalmente usando o software Maestro 3D Dental Studio. O ajuste das contenções foi avaliado posteriormente com um microscópio eletrônico de varredura. A precisão foi comparada entre três grupos, contenções projetadas digitalmente e aquelas impressas em alturas de camada de 50 µm e 100 µm, usando os testes ANOVA/Kruskal-Wallis. A confiabilidade e a consistência interna das medidas lineares foram avaliadas pelo coeficiente de correlação intraclasse (ICC com IC de 95%) e pelo alfa de Cronbach, respectivamente. Testes t pareados foram utilizados para comparar o ajuste de contenções impressas por prototipagem rápida, impressas em camadas de 100 µm e 50 µm. Além disso, o tempo de impressão de cada contenção foi registrado.

**Resultados:**

Não houve diferença estatisticamente significativa (valor de p > 0,05) entre a precisão das contenções impressas em 3D nas alturas de camada de 100 µm e 50 µm, com alta confiabilidade (ICC > 0,9) e alta consistência interna (alfa de Cronbach > 0,9). Na avaliação comparativa do ajuste, não foi observada diferença estatisticamente significativa (p > 0,05) entre as contenções impressas nas alturas de camada de 50 µm e 100 µm, exceto na borda labiogengival e no meio da borda incisal do incisivo central. O tempo necessário para a impressão das contenções na altura de 50 µm é maior do que o da altura da camada de 100 µm.

**Conclusão:**

Ambas as contenções de prototipagem rápida, impressas em camadas de 100 µm e 50 µm, se mostraram precisas para uso clínico, quando comparadas às contenções digitais de referência, com ajuste aceitável. Como o tempo necessário para imprimir contenções a 100 µm é menor do que as de camadas de 50 µm, é melhor usar camadas de 100 µm para a prototipagem rápida das contenções.

## INTRODUCTION

Retention is an essential part of orthodontic treatment. Increase in the esthetic demand of orthodontic appliances allows the orthodontist to use different commercially available thermoplastic materials to manufacture the clear retainers on either plaster models or Three-dimensional (3D) printed models.^1^ ^,^ ^2^ 3D printing is a method of reconstructing a three-dimensional structure based on digital computer-aided designed (CAD) data.^3^ 3D printing of the dental model is the first step towards the minimization of the geometric inaccuracies during impression collection.^4^ The conventional process of making thermoformed retainers involves dental impression, creating a plaster model, and then thermoforming a biocompatible plastic sheet. With the advent of extraoral / intraoral scanners, computer-aided designing (CAD) and 3D printing, a clear retainer can be accurately 3D printed. This process eliminates one extra step of thermoforming the plastic sheet on the plaster or direct printed model.^5^ In addition, it can eliminate the cumulative error introduced from analogue impression making and subsequent thermoforming work flow.^4^ The application of this technique in current orthodontic practice offers the potential for increasing efficiency and reducing waste.^6^

First, Nasef et al.^7^ reported this novel approach of direct printing of retainer where cone-beam computed tomography (CBCT) was used to generate standard tessellation language (STL) file of the retainer. Another study by the same investigators reported that the 3D-printed retainers were as accurate and reliable as the traditional vacuum-formed retainers. However, comparisons between the two retainers were made based on the linear measurements performed manually with the use of digital calipers.^8^

Very limited information is available about the dimensional accuracy of 3D printed retainers. Another study on dimensional accuracy of thermoformed retainers versus direct-printed retainers found greater discrepancies with direct printed retainer,^9^ whereas study by Jindal et al.^4^ showed that crown heights were more accurate with 3D printed retainers as compared to the thermoformed retainers. Dimensional accuracy of the direct printed retainer has been shown to be the effect of print angulation. Few studies showed no statistically significant difference in dimensional accuracy at different angulations of retainers on the build platform.^10^ ^,^ ^11^ Another most important factor influencing the dimensional accuracy of rapid prototyped retainer is the printing technique. Various 3D printing techniques available are stereolithography (SLA), digital light processing (DLP), polyjet photopolymerization (PPP) and fused deposition modeling (FDM). A previous study showed that, retainers fabricated by SLA, DLP, continuous DLP, and PPP technologies were shown to be clinically acceptable and accurate compared to the standard reference file. Based on both high precision and trueness, SLA and PPP printers yielded the most accurate retainers.^12^

A recent study found greater trueness and precision of direct-printed aligners as compared to the thermoformed aligners.^13^ Evaluation of the fit (i.e. gap) of the aligner over the 3D printed models for the purpose of anchorage was done using scanning electron microscopy (SEM), where Invisalign and CA-Clear Aligner both exhibited comparable fit on anchorage teeth.^14^ An another study used microcomputed tomography (micro-CT) images to check the aligner fit over resin cast, where the aligners presented good fit (especially in the anterior regions) which may affect their clinical efficacy and efficiency.^15^

Among the several factors affecting the accuracy of 3D printed retainers, one of the most important factors are print layer height. Print layer height represents the z-axis of printing perpendicular to the build platform. Higher resolution in the z-axis is a function of decreased layer height.^16^ Smaller distance between layers on diagonal surfaces allows finer details in the printed pieces. This greatly improves the surface finish. Therefore, smaller the layer height, the smoother the surface of the printed product.^17^ Study by Favero et al.^16^ and Loflin et al.^17^ showed that rapid prototyped model printed at 100 µm layer found to have least deviation from its reference digital file and clinically acceptable for the purposes of evaluation of treatment outcomes, diagnosis and treatment planning.

While previous studies focused on the accuracy of the 3D printed models as the effect of print layer height, there is no information in the literature about the effect of print layer height on the accuracy of direct printed retainers. Therefore, the present study conducted with the objective of comparative evaluation of the effect of different print layer height on the accuracy and fit of direct printed retainers.

## MATERIAL AND METHODS

### STUDY DESIGN

Post-treatment models of seven patients treated at the orthodontic division of a tertiary health care center were retrieved for scanning, followed by the design of retainers on these scanned digital models and subsequent rapid prototyping of the retainers. These retainers were rapid prototyped in two different layer heights (50 µm or 100 µm), with 3 retainers being produced for each layer height per model, resulting in a total of 6 retainers per model. So, considering the 7 models, the two layer heights and the 3 retainers per layer height, the final sample totaled 42 retainers. Therefore, this in-vitro study was conducted on 42 direct-printed retainers in the Division of Orthodontics and Dentofacial Deformities, Centre for Dental Education and Research, All India Institute of Medical Sciences, New Delhi. Prior approval was obtained from the Institutional Ethics Committee (reference number IECPG-645/25.11.2020) to carry out the study.

### SCANNING OF THE MODELS AND 3D PRINTING OF THE RETAINERS

**Figure 1:**
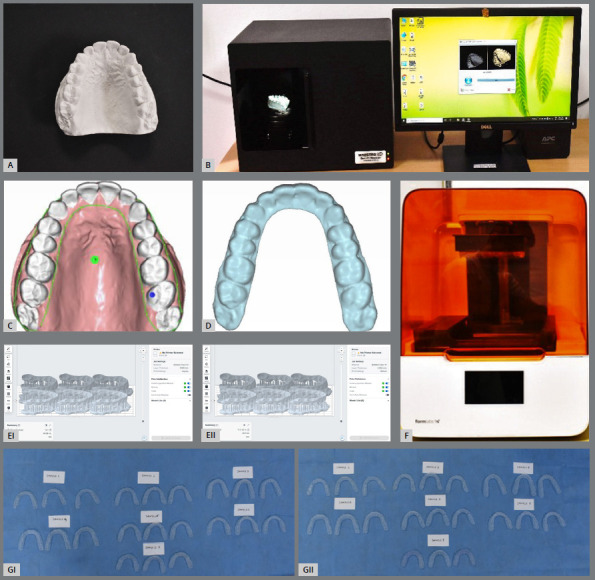
Overview of methodology: **A)** Plaster model -maxillary arch n=7; **B)** Scanning of dental model using Maestro 3D Desktop Scanner^®^ (AGE Solutions, Pisa, Italy); **C)** Designing of retainer over digital model; **D)** Photograph of retainer in STL file format; **E)** Orientation and support generation of the STL files of the retainers using PreForm Software (version 3.27.0)- **EI)** 50 µm layer height, **EII**) 100 µm layer height; **F)** Formlabs 3B Low force SLA printer; **G)** 3D printed retainers: **GI)** 50 µm layer height n=21 (3 retainers for each model), **G II)** 100 µm layer height n=21 (3 retainers for each model).

Seven Good quality post orthodontic plaster models were selected (Fig 1A). Plaster models were scanned by using Maestro 3D Desktop Scanner^®^ (AGE Solutions, Pisa, Italy) (Fig 1B). The scanned models were saved in the Standard Tessellation Language (STL) file format. Designing of the retainer over the scanned digital model was done using CAD software (Maestro 3D Dental Studio, AGE Solutions, Pisa, Italy) by selecting an option for virtual set up, then clear aligner on the toolbar. Thickness of the retainers was selected as 0.75 mm, without any offset. The outline of the dentition was drawn via selection of points contoured to the surface of the teeth (Fig 1C). The points were placed on the intended teeth for the future retainer and according to the desired extensions needed to avoid soft tissue impingement of the future retainer. Designed retainers were exported as STL file and saved (Fig 1D). The processed STL files were imported to PreForm software; version 3.27.0 (Formlabs Inc, Somerville, MA, USA). STL files were oriented horizontally or at 0° degree angulation with build platform without overlapping of the parts, with the impression surfaces facing away from the build platform, to ensure that supports will not be generated on these surfaces. Supports were generated using PreForm’s manual editing feature to strengthen the object being built up (Fig 1E). Parts were inspected to ensure there were no support touchpoints on intaglio surfaces. Material and the layer thickness (50 µm or 100 µm) were selected in the job setup option on the PreForm (Formlabs Inc, Somerville, MA, USA) software. The material used for 3D printing of the retainers was BioMed Clear V1 (Formlabs Inc, Somerville, MA, USA), a biocompatible, rigid, translucent for long term skin and mucosa contact (> 30 days). It uses the support material same as the main material used for printing the desired object. As the print job was completed by the Formlabs printer 3B (Formlabs Inc, Somerville, MA, USA) (Fig 1F), the printing time was reflected on the printer’s screen for each slot. Total time required for printing was calculated for both groups of retainers (i.e.50 µm and 100 µm layer height) (Table 1). Thus, we obtained the rapid prototyped retainers (Fig 1G) of 0.75 mm thickness printed at 50 µm and 100 µm layer height increments. Supports were cut from the build platform and detached from the retainers. Retainers were rinsed with Isopropyl Alcohol (IPA, 96% or higher) for 15 minutes, then soaked in fresh Isopropyl Alcohal for 5 minutes, which cleaned the parts and removed the excess liquid resin. IPA was blown out by using compressed air or object can be left for 30 minutes for air drying. After that, retainers were placed in form-cure for 20 minutes where they got exposed to light to achieve optimal mechanical properties.

**Table 1:** Comparison of 3D printed retainers printed at 100 µm and 50 µm in terms of amount of material used and printing time.

No. of retainers	3D printed retainers at 100 µm		3D printed retainers at 50 µm	
	Volume of resin used (ml)	Time for printing (minutes)	Volume of resin used (ml)	Time for printing (minutes)
21	58.75 ml	477 minutes	61.11 ml	738 minutes
1	2.79 ml	22.71 minutes	2.91 ml	35.14 minutes

### EVALUATION OF THE ACCURACY OF THE RETAINERS

Measurements were made on the digitally designed retainers and rapid prototyped retainers by using Maestro 3D Dental Studio software (AGE Solutions, Pisa, Italy) and digital caliper with an accuracy of 0.01 mm respectively. The examiner was trained before starting the measurement and same examiner performed all the measurement.

**Figure 2:**
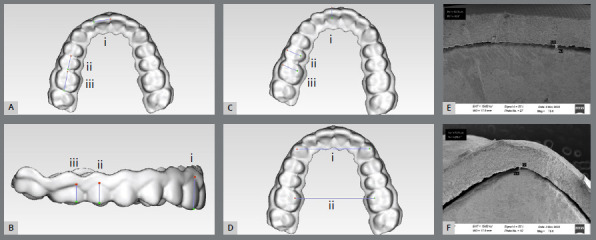
A) i Central incisor width; ii second premolar width; iii First molar width; B) i Central incisor height; ii Second premolar height; iii First molar height; C) i Central incisor thickness; ii Second premolar thickness; iii First molar thickness; D) i Intercanine width; ii Intermolar width; SEM image: E) Interaction between retainer and tooth at gingival margin; F) Interaction between retainer and tooth at cusp tip.

The parameters used for measuring the accuracy were as follows:

» Central incisor width: measured from mesial contact point to distal contact point of central incisor (Fig 2A, i).
» Premolar width: measured from mesial contact point to distal contact point of second premolar (Fig 2A, ii).
» Molar width: measured from mesial contact point to distal contact point of first molar (Fig 2A, iii).
» Central incisor height: measured from gingival zenith to incisor edge of central incisor (Fig 2B, i).
» Premolar height: measured from gingival zenith to buccal cusp tip of second premolar (Fig 2B, ii).
» Molar height: measured from gingival zenith to mesio-buccal cusp tip of first molar (Fig 2B, iii).
» Central incisor thickness: measured from mid points of labial and lingual surface of central incisor (Fig 2C, i).
» Premolar thickness: measured from buccal cusp tip to palatal cusp tip of second premolar (Fig 2C, ii).
» Molar thickness: measured from mesio-buccal cusp tip to mesio-palatal cusp tip of first molar (Fig 2C, iii).
» Inter-canine width: measured from the cusp tip of the right-side canine to the cusp tip of the left-side canine (Fig 2D, i).
» Inter-molar width: measured from mesio-palatal cusp tip of right-side 1^st^ molar to mesio-palatal cusp tip of left side 1^st^ molar (Fig 2D, ii).

### EVALUATION OF THE FIT OF THE 3D PRINTED RETAINERS

Fit of the rapid-prototyped retainers was evaluated by using Scanning Electron Microscopy (SEM). This was done by sectioning the 3D printed retainers adapted over their respective plaster models. Sections were made at the central incisor, canine and first molar region such that the maximum buccolingual width of the tooth was obtained and the observable surface was perpendicular to the electron beam. The prepared specimen was loaded in Scanning electron microscopy (SEM) machine and exposed to the electron beam which was accelerated at the voltage of 15 kV and a distance of 14.5 mm. This shows the surface topography of the tooth segment and the retainer over it along with the microphotograph of the same. The fit of the rapid prototyped retainers printed at two different layers height (50 µm and 100 µm) over their respective models were analyzed by measuring the distance between occlusal surface of reference model and intaglio surface of 3D printed retainers (Fig 2E,F).

### STATISTICAL ANALYSIS

All the data were analysed using STATA software (16.1, Stata Corp LLC, College Station, TX, USA). The P-value for statistical significance for linear measurements was set as ≤ 0.05 at the confidence interval of 95%.

One-way analysis of variance (ANOVA) tests/ Kruskal-Wallis tests were used to check the accuracy by evaluating the variation amongst the linear measurements of 3 groups (Rapid-prototyped retainers printed in 50 µm and 100 µm layer height and the digital retainers).

Reliability and internal consistency of all the linear measurements made on the digital retainers and on the rapid-prototyped retainers printed in 50 µm and 100 µm layer height for evaluating the 3-dimensional accuracy were demonstrated by Intraclass Correlation Coefficient (95% Confidence Interval) and Cronbach’s Alpha value.

Paired t-test was used to compare the fit of rapid-prototyped retainers printed at 100 µm and 50 µm layer height adapted over plaster models. Coefficient of variance was calculated to check the proportion of variability.

## RESULTS

The results were formulated based on the comparison of linear measurements made on the digital retainers and 3D printed retainers at 100 µm and 50 µm, in all 3-dimension at the predetermined landmarks.

One-way analysis of variance ANOVA/Kruskal-Wallis tests were used to compare the accuracy by checking the variation amongst linear measurement of the 3 groups (digital retainers, rapid-prototyped retainers printed at 100 µm and 50 µm layer height) showed statistically insignificant difference (p>0.05) (Table 2).

**Table 2:** One-way ANOVA/Kruskal-Wallis* test to compare 3-Dimensional accuracy of the rapid-prototyped retainers printed at 50 µm and 100 µm layer height with digital retainers (STL).

Variables	STL	50 Micron	100 Micron	p-value
	Mean±SD/	Mean±SD/	Mean±SD/	
	Median (IQR) *	Median (IQR) *	Median (IQR) *	
CI width	9.16±0.53	9.17 ±0.49	9.19±0.50	0.992
CI height*	8.81 (8.32-10.14) *	8.55 (8.27-10.13) *	8.81 (8.25-10.16) *	0.872
CI thickness	4.72±0.26	4.59 ±0.17	4.72±0.31	0.549
PM width	6.93±0.37	6.98 ±0.36	6.90±0.38	0.922
PM height	4.91±0.55	5.11 ±0.46	4.95±0.54	0.760
PM thickness*	6.20 (6.16-6.38) *	6.31 (6.16-6.48) *	6.14 (6.08-6.36) *	0.495
molar width	10.45±0.48	10.41 ±0.52	10.46±0.53	0.982
molar height	5.02±0.70	5.09 ±0.68	4.99±0.70	0.966
molar thickness	7.09±0.30	7.05 ±0.30	7.04±0.30	0.957
ICW	35.33±1.68	35.33±1.65	35.09±1.70	0.953
IMW	37.40±2.26	36.91±2.66	36.89±2.47	0.910

*Median and IQR are reported and Kruskal-Wallis test was performed.

CI: Central Incisor. PM: Premolar. ICW: Intercanine width. IMW: Intermolar width. SD: Standard deviation. IQR: Inter quartile range.

The reliability and internal consistency of the measurements are excellent with Intraclass Correlation Coefficient (ICC) > 0.9 and Cronbach’s Alpha > 0.9 in most of the variables except central incisors thickness and molar thickness among the 3 groups as well as in between the digital retainers and 3D printed retainers at 50 µm layer height. Whereas, the reliability and internal consistency of the measurements are excellent for all the variables with Intraclass Correlation Coefficient (ICC) > 0.9 and Cronbach’s Alpha > 0.9 between the digital retainers and 3D printed retainers at 100 µm layer height (Table 3).

**Table 3:** Intraclass Correlation Coefficient, Cronbach’s Alpha, for linear measurements made on retainers in each layer height and STL for each model.

Variable	STL, 50 µm and 100 µm		STL and 50 µm		STL and 100 µm	
	ICC (95% Confidence Interval)	Cronbach’s Alpha	ICC (95% Confidence Interval)	Cronbach’s Alpha	ICC (95% Confidence Interval)	Cronbach’s Alpha
CI width	0.979 (0.923,0.996)	0.993	0.986 (0.921,0.998)	0.993	0.986 (0.920,0.998)	0.993
CI height	0.970 (0.896,0.994)	0.990	0.952 (0.752,0.992)	0.976	0.996 (0.980,0.999)	0.998
CI thickness	0.450 (0.025,0.854)	0.711	0.009 (0.702,0.711)	0.018	0.964 (0.808,0.994)	0.982
PM width	0.97 (0.902,0.995)	0.991	0.957 (0.771,0.992)	0.978	0.957 (0.771,0.992)	0.978
PM height	0.893 (0.667,0.979)	0.962	0.821 (0.273,0.967)	0.902	0.940 (0.695,0.989	0.969
PM thickness	0.916 (0.730,0.984)	0.971	0.900 (0.530,0.982)	0.947	0.977 (0.872,0.996)	0.988
Molar width	0.987 (0.952,0.998)	0.996	0.990 (0.941,0.998)	0.995	0.984 (0.913,0.997)	0.992
Molar height	0.991 (0.966,0.998)	0.997	0.996 (0.978,0.999)	0.998	0.985 (0.916,0.997)	0.992
Molar thickness	0.871 (0.611,0.974)	0.953	0.794 (0.198,0.961)	0.885	0.977 (0.874,0.996)	0.988
ICW	0.981 (0.930,0.996)	0.993	0.976 (0.866,0.996)	0.988	0.978 (0.877,0.996)	0.989
IMW	0.984 (0.942,0.997)	0.995	0.907 (0.559,0.983)	0.951	0.964 (0.809,0.994)	0.982

IG1: Labio-gingival border of central incisors. IE: Mid of incisal edge of central incisors. IG2: Linguo-gingival border of central incisors. CG1: Labio-gingival border of canine. CCT: Cusp tip of canine. CG2: Linguo-gingival border of canine. MG1: Labio-gingival border of first molar. MCT1: Mesio-buccal cusp tip of first molar. MCT2: Mesio-palatal cusp tip of first molar. MG2: Linguo-gingival border of first molar. CV: Coefficient of variance.

Paired t-test was used to compare the fit of rapid-prototyped retainers printed at 50 µm and 100 µm layer height based on the discrepancy (distance between occlusal surface of plaster model and intaglio surface of 3D printed retainer). There was statistically no significant difference observed for most of the parameters with p-value>0.05 except for the measurements made at mid of the incisal edge of central incisor (0.28±0.13 mm and 0.16±0.14 mm at 50 µm and100 µm layer height respectively) and labio-gingival border of central incisor (0.18±0.098 mm and 0.087±0.042 mm at 50 µm and 100 µm layer height respectively) which showed statistically significant difference with p-value <0.05. Proportion of variability was assessed, showing range from 25% to 100% (Table 4).

**Table 4:** Paired t-test to compare the fit of rapid-prototyped retainers printed at 50µ and 100µ layer height over the plaster models.

Parameters	Retainers printed at 50µ		Retainers printed at 100µ		p-value
	Mean ±SD	CV(%)	Mean ±SD	CV(%)	
IG1	180.42±98.90	54.8	87.25±42.70	48.9	0.046
IE	289.37±138.86	47.9	163.90±141.85	86.5	0.036
IG2	266.42±97.54	36.6	277.51±101.89	36.7	0.654
CG1	214.11±133.97	62.5	141.93±135.68	95.5	0.443
CCT	147.72±103.28	69.9	126.87±85.71	67.5	0.695
CG2	202.3±59.59	29.4	148.90±88.44	59.3	0.113
MG1	119.67±123.78	103.4	51.43±31.96	62.1	0.211
MCT1	180.62±124.91	69.1	146.45±161.75	110.4	0.713
MCT2	157.68±80.63	51.1	168.52±102.88	61.0	0.811
MG2	161.41±65.31	40.4	96.05±71.34	74.2	0.166

IG1- Labio-gingival border of central incisors. IE- Mid of incisal edge of central incisors. IG2- Linguo-gingival border of central incisors. CG1- Labio-gingival border of canine. CCT-Cusp tip of canine. CG2- Linguo-gingival border of canine. MG1- Labio-gingival border of first molar. MCT1-Mesio-buccal cusp tip of first molar. MCT2-Mesio-palatal cusp tip of first molar. MG2- Linguo-gingival border of first molar. CV- Coefficient of variance.

## DISCUSSION

This study was planned as an exploratory study to check the fit along with accuracy of the rapid-prototyped retainers. The choice of sample size was based on previous study by Cole et al.^9^ as a reference and it was decided to use more than twice the number of samples of the previous study to ensure the robustness of the results.

Many factors should be thoughtfully considered when selecting and applying a 3D printing in orthodontics. Variables to consider includes types of printing technique, print resolution (layer height), print orientation, post-processing steps, printing time, cost (including maintenance and materials). Many of these variables could affect the accuracy of printed parts.^16^

First important factor is printing technique or printer type, differences between printer types are more convoluted and often depend on proprietary design, engineering, optics and material chemistry. A recent study by Naeem et al.^12^ stated that, retainers fabricated by SLA, DLP and PPP technologies were shown to be clinically acceptable, but based on the precision and trueness, SLA and PPP printers yielded the most accurate retainers. Whereas, a study by Favero et al.^16^ observed that DLP printers are the most accurate as compared to PPP and SLA printers. Similarly, Zhang et al.^18^ also stated that at a given layer thickness of 100 µm, the printing accuracy of DLP printers are superior to those of SLA printers.

Although, there is controversy still going on regarding the accuracy of printer type/printing technologies with the advancement and upgradation. A recent study by Grassia et al.^19^ on the accuracy of 3D printed models for aligners production showed lower trueness error with SLA 3D printer compared to DLP but as compared to SLA, DLP showed lower precision error. The greater accuracy of the rapid prototyped retainers in our study could be because of the printing technique used. LFS (Low Force Stereolithography) printer, which is an advanced form of SLA (Stereolithography) printer yielded the accurate retainers. It was introduced to overcome the shortcomings of SLA printers which exert significant force while printing. LFS 3D printing uses a flexible resin tank to significantly reduce peel forces during printing and a Light Processing Unit (LPU), a custom-designed enclosed optics engine used to produce consistent and accurate prints. Introduction of Low Force Stereolithography (LFS) drastically reduced the forces exerted on parts during the print process allowing for light-touch support structures that tear away easily. 

Thereby, this technique is able to deliver great surface quality and print accuracy.^20^ The superiority of SLA printer over a DLP printer could be because of the reason that SLA 3D printers use a laser beam to cure the resin and it is not dependent by x-y resolution; instead, DLP printers build the object by curing the resin layer by layer and could generate subtle artifacts at layer edges that look like “staircase steps” due to the pixelated illumination generated from the light source (projector or LCD screen for LCD 3D printers).^19^

The second most important factor is the print layer height. As discussed by Favero et al.^16^ and Loflin et al.^17^, the print layer height represents the z-axis of printing perpendicular to the build platform. Higher resolution in the z-axis is a function of decreased layer height. However, although a smaller layer height (higher resolution) will likely allow for more detail in a printed pieces and an improved surface finish, it does not always mean that the printed piece is more accurate than those printed with larger layer settings.

The accuracy checked between the gold standard digital retainers and the 3D printed retainers showed statistically insignificant difference with p>0.05 for the linear measurement made in 3 planes involving individual tooth measurements and arch dimension. A discrepancy of up to 0.25 mm was observed between the 3D printed retainers and reference digital file except for the intermolar width at 3D printed retainers both at 100 µm and 50 µm layer height. This finding coincides with the results of Naeem et al.^12^ where a deviation within the range of 0.25 mm between the printed retainers and reference digital retainers indicated accuracy and clinical acceptability. The results also found to be similar to the study by Williams et al.^11^ where the 3D printed retainers were observed to be with in 0.25 mm deviation with the digital reference retainers.

The reliability and internal consistency of the rapid prototyped retainers were evaluated by the linear measurements made on rapid prototyped retainers printed at 100 µm and 50 µm layer height were found to be excellent for all the variables with Intraclass Correlation Coefficient (ICC) > 0.75 and Cronbach’s Alpha > 0.9. These finding coincides with the results of Cole et al.^9^ and Williams et al.^11^ where, the internal consistency was 0.88 and > 0.75 respectively.

Regarding the fit of 3D printed retainers, in the present study we evaluated the fit of retainers by preparing the specimens under laboratory condition, microphotographed using Scanning electron microscope and by measuring the distance between occlusal surface of plaster model and intaglio surface of 3D printed retainers. Total 140 measurements were done and all the measurements were found to be within the range of 0.5 mm which has previously been accepted to be clinically sufficient.^21^ ^,^ ^22^ Among these 140 measurements, only 30 were >0.25 mm, rest 110 were < 0.25 mm. This finding was similar to the results of a study by Cole et al.^9^, where fit of the 3D printed retainers were evaluated and it was found that all the retainers showed deviation within 0.5 mm, however the study by Cole et al.^9^ used a software to superimpose the digital image of 3D printed retainers and their original reference model.

The fit was only judged to be good or acceptable after manually seating the printed retainers over the reference model. In the literature, there is no information on the maximum acceptable distance between the retainers and their master cast to be accurate enough for the clinical use.^9^ Based on the study by Boyd and Waskali^23^ a minimum of 0.15 to 0.25 mm distance needs to be exist between the cast and the appliance for an aligner to cause tooth movement. However, a precise fit of the appliance is essential for successful retention.^24^ In our study, most of the sample were found to be in the range of 0.25-0.50 mm in 7 parameters i.e. mid of the incisal edge, linguogingival border of incisor, labio-gingival border of canine, cusp tip of canine, linguo-gingival border of canine, mesio-lingual cusp of molar and mesio-palatal cusp of molar. There is no statistically significant difference in the fit of retainers printed at 50 µm and 100 µm layer height over their respective plaster models except at middle of the incisal edge and at the labio-gingival border of incisor.

Biocompatibility is a very important factor when considering 3D printed appliances, especially for applications where the appliance comes in contact with the body tissues. In the present study, BioMed Clear (Formlabs Inc, Somerville, MA, USA) resin was used which is a biocompatible, rigid, translucent material for long term skin and mucosa contact (> 30 days). This USP (United States Pharmacopoeia) Class VI certified material is suitable for applications that require wear resistance and low water absorption over time.^25^ In most of the studies pertaining to 3D printed retainers/aligners,^9^ ^,^ ^11^ ^,^ ^26^ Dental LT Clear (Formlabs Inc, Somerville, MA, USA) resin was used having all most all the physical (i.e. Color and translucency) and mechanical properties (i.e. tensile, flexural, and hardness properties) are similar to BioMed Clear.^25^ The process of development of material, suitable for orthodontic application is still ongoing to produce a clear, esthetic, biocompatible material which remain stable at body temperature, strong enough to withstand the occlusion forces and which does not break down over time. A photopolymerizable polyurethane (Tera Harz TC-85DAP 3D Printer UV Resin^®^; Graphy Inc., Seoul, Korea) specifically indicated to print aligners has become available. In a recent study by Koenig et al.^13^ this material was used for direct printed aligners which showed greater trueness and precision than thermoformed aligners.

Since, the study was carried out as an exploratory study, the results with this much sample size were found to be clinically acceptable in terms of accuracy i.e. deviation within 0.25 mm and fit i.e. deviation within 0.5 mm (highest mean 0.29±0.14 mm at the incisal edge) of the rapid prototyped retainers. In addition, all the retainers printed at 50 µm and 100 µm layer height showed high reliability and internal consistency with ICC and Cronbach’s Alpha > 0.9 (Table 3) among the measurements. This close agreement of measurements indicates that 3D printed retainers printed at 50 µm and 100 µm layer height, both are equally accurate as their reference digital retainers and can be use clinically for the purpose of retention.

The discussion has been expanded on the implications of findings for clinical decision-making.

As the previous studies^16^ ^-^ ^18^ showed, the print layer height and the number of layers required to print an object may affect the clinical accuracy, printing speed and printing time, hence the present study was performed to identify a more efficient and economical approach that can influence the clinical decision-making process while implementing the 3D printed retainers in day-to-day clinical practice. In orthodontics, economic analysis is important as a basis for decision making in planning and management of dental care and allocating resources. Economic evaluations with a societal perspective include calculations of direct and indirect costs.^27^ Use of only one printer (i.e. SLA) in this study shows clinically acceptable accuracy and fit with both the layer heights (i.e. 50 µm and 100 µm), while the amount of material used for printing a single retainer was almost equal with each of the print layer height (Table 1). There was no difference in direct cost (i.e. cost of material and the cost of equipment) for 3D printing. But as the time required for printing retainers at 100 µm is less than that of 50 µm layer height, it affects the indirect cost (i.e. electricity consumed) of 3D printing. So, it is recommended to use 100 µm layer height to print the retainers for the orthodontists looking to implement 3D printing for the retainers with clinically acceptable accuracy and fit.

The limitation of the present study was using smaller sample size, one specific printer model and resin type, which may limit the generalizability of the results. Thus, future studies can evaluate the effect of print layer height on the accuracy of 3D printed retainers from different 3D printing techniques with different resin types suitable for orthodontic applications with larger sample size.

## CONCLUSION

The findings of the study concluded as follows: 

There is no significant difference in the accuracy between the rapid prototyped retainers printed at 50 µm and 100 µm layer height using LFS printer for the linear measurements made in comparison with reference digital retainers indicating no effect of print layer height on the accuracy of 3D printed retainers. 

There was no significant difference found in the fit of the retainers printed at 50 µm and 100 µm layer height over the plaster models using Scanning Electron Microscope (SEM) study.

Rapid-prototyped retainers printed at 100 µm and 50 µm layer heights, both were found to be accurate for clinical use when compared to reference digital retainers with acceptable fit. As the time required for printing retainers at 100 µm is less than that of 50 µm layer height, it is better to use 100 µm layer height for rapid prototyping of the retainers.
